# Draft Genome Sequence of *Rubricoccus marinus* SG-29^T^, a Marine Bacterium within the Family *Rhodothermaceae*, Which Contains Two Different Rhodopsin Genes

**DOI:** 10.1128/genomeA.00990-17

**Published:** 2017-09-21

**Authors:** Yu Nakajima, Susumu Yoshizawa, Sanghwa Park, Yohei Kumagai, Shu-Kuan Wong, Yoshitoshi Ogura, Tetsuya Hayashi, Kazuhiro Kogure

**Affiliations:** a Laboratory of Marine Microbiology, Department of Marine Ecosystem Dynamics, Division of Marine Life Science, Atmosphere and Ocean Research Institute, the University of Tokyo, Kashiwa, Chiba, Japan; b Center for Earth Surface System Dynamics, Atmosphere and Ocean Research Institute, the University of Tokyo, Chiba, Japan; c Bacterial Resources Research Division, Freshwater Bioresources Research Bureau, Nakdonggang National Institute of Biological Resources (NNIBR), Gyeongsangbuk-do, Republic of Korea; d Department of Bacteriology, Faculty of Medical Sciences, Kyushu University, Fukuoka-shi, Fukuoka, Japan

## Abstract

Here, we report the draft genome sequence of *Rubricoccus marinus* SG-29^T^, a bacterium isolated from the western North Pacific Ocean. *R. marinus* SG-29^T^ possesses two different types of rhodopsin genes and belongs to the family *Rhodothermaceae*, with which halophilic, thermophilic, and marine bacteria are associated.

## GENOME ANNOUNCEMENT

*Rubricoccus marinus* SG-29^T^ is an aerobic Gram-negative bacterium belonging to the family *Rhodothermaceae*, in the phylum *Bacteroidetes*. This family consists of 7 described genera and 12 described species. SG-29^T^ was the first ocean surface isolate discovered in the family, which was obtained from seawater at a depth of 50 m in the western North Pacific Ocean (30°40′N, 138°00′E) (1).

The genomic DNA of strain SG-29^T^ was extracted using phenol-chloroform and ethanol precipitation (2). An 800-bp paired-end library and an 8-kb mate pair library were prepared using the Nextera XT DNA library preparation kit (Illumina) and Nextera mate pair sample preparation kit (Illumina), respectively. Each end of the libraries (300 bp) was sequenced on the MiSeq instrument with the MiSeq Reagent kit version 3 (Illumina). Genome assembly was performed using *Platanus* version 1.2.4 (3). The assembled sequences were annotated using the NCBI Prokaryotic Genome Annotation Pipeline (PGAP) (4) and reviewed with Rapid Annotations using Subsystems Technology (RAST) version 2.0 (http://rast.nmpdr.org). The draft genome sequence of *R. marinus* SG-29^T^ consists of 15 scaffolds (total length, 4,430,441 bp; *N*_50_, 167,811 bp), with a median read coverage of 58× and a G+C content of 69.0 mol%. This G+C content is higher than the average G+C content of isolates within the phylum *Bacteroidetes* (29.5% to ~64.3%), to which the family *Rhodothermaceae* belongs (5).

PGAP identified 3,773 genes, including 3,721 protein-coding sequences (CDSs), 46 tRNA and 3 noncoding RNA genes, and 51 pseudogenes. RAST annotation also identified 3,847 CDSs. Genome sequence analysis revealed that *R. marinus* SG-29^T^ has two predicted rhodopsin genes. Phylogenetic analysis revealed that one rhodopsin gene belongs to the xenorhodopsin clade (6), while the other is closely related to freshwater cyanobacterial halorhodopsins (7, 8). The results of RAST annotation showed that strain SG-29^T^ possesses nine genes related to the “phage, prophage, transposase” category. Among these, 2 and 6 genes, respectively, were classified in the subsystems “integrons” and “*Listeria* pathogenicity island,” which are related to horizontal gene transfer. The numbers of the genes for this category are higher than those for other members within the family *Rhodothermaceae* (1 in *Rhodothermus marinus* DSM 4252^T^ [GenBank accession number NC_013501], 1 in *Rhodothermus profundi* DSM 22212^T^ [accession number NZ_FRAU00000000], 0 in *Salinibacter ruber* DSM 13855^T^ [accession number NC_007677], and 3 in *Salisaeta longa* DSM 21114^T^ [accession number NZ_ATTH00000000]). As the estimated genome size of SG-29^T^ is significantly larger than those of strains *R. marinus* DSM 4252^T^ (3.33 Mbp), *R. profundi* DSM 22212^T^ (3.14 Mbp), *S. ruber* DSM 13855^T^ (3.71 Mbp), and *S. longa* DSM 21114^T^ (3.40 Mbp), the higher number of strain SG-29^T^ genes in this category may suggest more frequent gene acquisition by horizontal gene transfer.

Because the family *Rhodothermaceae* includes marine bacteria, halophilic bacteria, and thermophilic bacteria, a novel genome sequence of a member from this family will facilitate our understanding of the physiology and mechanisms of how each group has adapted to either extreme or marine environments.

### Accession number(s).

The whole-genome shotgun projects of *Rubricoccus marinus* SG-29^T^ have been deposited in DDBJ/EMBL/GenBank under the accession number MQWB00000000. The version described in this paper is the first version, MQWB01000000.
